# Photoluminescence of spray pyrolysis deposited ZnO nanorods

**DOI:** 10.1186/1556-276X-6-359

**Published:** 2011-04-21

**Authors:** Erki Kärber, Taavi Raadik, Tatjana Dedova, Jüri Krustok, Arvo Mere, Valdek Mikli, Malle Krunks

**Affiliations:** 1 Department of Materials Science, Tallinn University of Technology, Ehitajate tee 5, 19086 Tallinn, Estonia; 2 Centre for Materials Research, Tallinn University of Technology, Ehitajate tee 5, 19086 Tallinn, Estonia

## Abstract

Photoluminescence of highly structured ZnO layers comprising well-shaped hexagonal rods is presented. The ZnO rods (length 500-1,000 nm, diameter 100-300 nm) were grown in air onto a preheated soda-lime glass (SGL) or ITO/SGL substrate by low-cost chemical spray pyrolysis method using zinc chloride precursor solutions and growth temperatures in the range of 450-550°C. We report the effect of the variation in deposition parameters (substrate type, growth temperature, spray rate, solvent type) on the photoluminescence properties of the spray-deposited ZnO nanorods. A dominant near band edge (NBE) emission is observed at 300 K and at 10 K. High-resolution photoluminescence measurements at 10 K reveal fine structure of the NBE band with the dominant peaks related to the bound exciton transitions. It is found that all studied technological parameters affect the excitonic photoluminescence in ZnO nanorods.

PACS: 78.55.Et, 81.15.Rs, 61.46.Km

## Introduction

ZnO is a semiconductor material for various photonic and electrical applications. ZnO shows a unique set of physical and chemical properties, such as a wide band gap (3.37 eV), large exciton binding energy (60 meV) at room temperature, radiation hardness [1], piezoelectricity and photoelasticity [2] and surface chemistry sensitive to environment. Zinc oxide nanostructured layer comprizing nanorods, further denoted as ZnO nanorod layer (ZnO_NRL_), is a material with large effective surface area, suitable for short-wavelength devices, such as ultraviolet (UV) light-emitting diodes (LED) [3,4], UV nanolaser arrays [5], UV photodetectors [6], field emitters [7], UV protectors-filters [8], and chemical sensors [9,10]. As a passive layer with light-trapping properties, ZnO_NRL _can be used as an antireflection coating on silicon solar cells [11], surface-enhancing window layer in the second generation solar cells with extremely thin inorganic absorber layer (ETA) [12] or with dye-sensitized solar cells (DSSC) [13]. The chemical spray pyrolysis (CSP)-deposited ZnO_NRL _was used in ETA solar cells showing energy conversion efficiency of 4.2% [14].

For many of such devices, a large-scale, low-cost fabrication of high optical and crystalline quality ZnO is desirable. Low-temperature chemical synthesis methods can provide large scale and low-cost fabrication. Photoluminescence (PL) is a very sensitive and an effective method to identify the dominant recombination mechanism and defects in materials. However, according to PL study, the as-deposited ZnO_NRL _grown via a low-temperature wet-chemical methods (chemical bath, electrodeposition, hydrothermal growth) do not show a high excitonic to visible emission intensity ratio at 300 K (room temperature), indicating a high defect concentration in these samples [15-21]. The PL properties of ZnO_NRL _deposited by the wet-chemical methods can be improved by post-growth annealing at high temperatures of 200-850°C in forming gas or vacuum environment [15,17-19,21].

In this study, strong excitonic PL is observed in the as-deposited ZnO_NRL_, grown via CSP at growth temperatures up to 550°C from zinc chloride (ZnCl_2_) solutions. CSP technique is a template- and catalyst-free method, allowing fast and low-cost deposition of ZnO_NRL _[22]. According to XRD, ZnO_NRL _comprise *c*-axis (002)-oriented, hexagonal rods of pure ZnO wurtzite phase with aspect ratio up to 30 [23,24]. The aim of this work is to study the variation in the PL response due to different growth parameters used for the CSP-deposited ZnO_NRL_. As a result, the PL properties were found to depend on the growth temperature, substrate type, spray rate, and the solvent type. The correlation between the electrical and the photoluminescence properties of the CSP-deposited ZnO_NRL _were reported earlier [25]. Our previous studies on the CSP-deposited ZnO_NRL _were focused on the development of the ZnO_NRL _[22-24], no specific study on the PL of the CSP-deposited ZnO_NRL _was reported until now.

## Experimental

The ZnO_NRL _were deposited by pneumatic CSP method in air at growth temperatures (*T*_G_) of 480°C, 530°C, 550°C using zinc chloride (ZnCl_2_) precursor solutions. Two kind of substrates were used: soda-lime glass (SGL) and commercial indium tin oxide-covered glass (ITO/SGL). The growth temperature *T*_G _was controlled through the temperature of a molten tin bath used to obtain uniform heating of the substrate. Other deposition parameters were varied in the following: Three discrete values were used for the spray rate (*v*): 1.2, 2.2, and 6.2 ml/min. Two kinds of solvents for ZnCl_2 _were used: H_2_O and alcoholic solvent (H_2_O + ethanol, in ratio of 2:3 by volume), the volume of spray solution was 50 ml. The concentration of ZnCl_2 _in the spray solution was kept constant in the presented series ranging from 0.05 mol/L ( Effect of the substrate on PL properties of ZnO_NRL _and Effect of the spray rate on PL properties of ZnO_NRL _sections) to 0.1 mol/L (General properties of ZnO_NRL_, Effect of the growth temperature on PL properties of ZnO_NRL_, and Effect of solvent type on PL properties of ZnO_NRL _sections). The acidity of the solution was kept at pH = 5. The studied samples were as grown. XRD measurements were performed on a Rigaku Ultima IV diffractometer with Cu K_α _radiation (*λ *= 1.5406 Å) using the silicon strip detector D/teX Ultra.

The photoluminescence (PL) measurements of ZnO_NRL _were made at room temperature (*T *= 300 K) and at *T *= 10 K in a closed-cycle He cryostat (Janis). He-Cd laser (325 nm) was used as an excitation source, the excitation intensity was approximately 0.5 mW/mm^2^. The luminescence emission in the energy region of 1.45-3.45 eV was dispersed by a computer-controlled Carl Zeiss SPM-2 monochromator (*f *= 0.4 m) equipped with a prism and detected by FEU-79 photomultiplier with a lock-in amplifier. High-resolution LabRam Horiba Yvon HR 800 spectrometer and CCD-detector were used in the region of 3.30-3.40 eV for a closer study of the PL of ZnO_NRL _in UV-region. The same apparatus was used for the Raman spectroscopic studies. Zeiss EVO-MA15 apparatus was used for the scanning electron microscopic (SEM) and the energy dispersive spectroscopic (EDS) study of the ZnO_NRL_. The apparatus was equipped with the Oxford Instruments PentaFet x3 spectrometer using the INCA Energy EDS system at accelerating voltage of 7 kV.

## Results and discussion

### General properties of ZnO_NRL_

**Figure 1:**
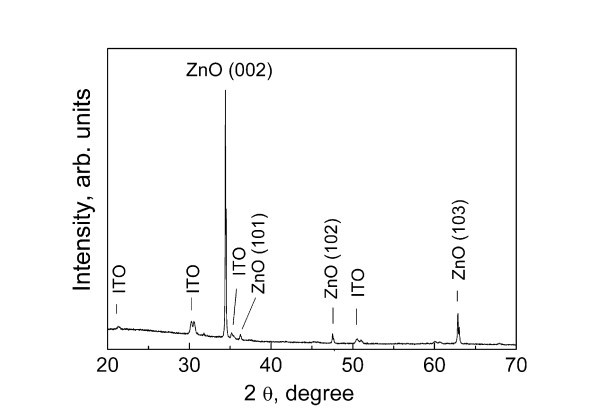
**XRD pattern of ZnO nanorod layer deposited by chemical spray pyrolysis**. The layer was deposited onto ITO/SGL substrate at growth temperature of 550°C, using aqueous solution. SEM image of the corresponding ZnO nanorod sample is presented in Figure 3a.

**Figure 2:**
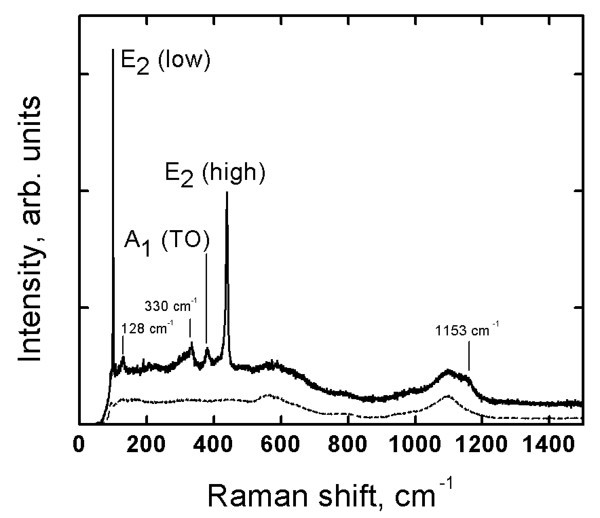
**Raman spectrum of ZnO nanorod layer deposited by chemical spray pyrolysis**. The layer was deposited onto ITO/SGL substrate at growth temperature of 550°C, using aqueous solvent. SEM image of the corresponding ZnO nanorod sample is presented in Figure 3a. The Raman spectrum of the ITO/SGL substrate is shown with the dashed line.

Typical XRD pattern of sprayed ZnO_NRL _is presented in Figure 1. According to XRD, the as-grown ZnO_NRL _are highly *c*-axis-oriented hexagonal (wurtzite) ZnO structures. The Raman spectrum of as-deposited ZnO_NRL _is presented in Figure 2. Raman peaks located at 99 and 438 cm^-1 ^with a fitted excitonic peak widths (FWHM) of 1.2 and 6.1 cm^-1^, respectively, are dominant ones. Raman peaks at 99, 438, and 379 cm^-1 ^are attributed to the E_2_(low), E_2_(high), and A_1_(TO) Raman modes of wurtzite phase of ZnO, respectively [26]. The Raman peak at approximately 580 cm^-1 ^which is correlated to V_O _and/or Zn_i _defects [27] is not observed. The peaks at 128, 330, and 1,153 cm^-1 ^are due to the second order or multiple phonon scattering of the Raman modes of the ZnO wurtzite structure, observed by other authors as well [28]. The sharp Raman peaks characteristic of the wurtzite phase and the absence of defect-induced Raman peaks is an indication of a high-quality crystalline material. According to EDS analysis, the O/Zn atomic ratio in ZnO_NRL _is ca. 1.5. The excess of oxygen (compared to the stoichiometric ZnO) as well as the presence of In, Sn, and Si signal in the EDS spectra (not presented), originates from the ITO/SGL substrate. Other elements are not detected by the EDS.

**Figure 3:**
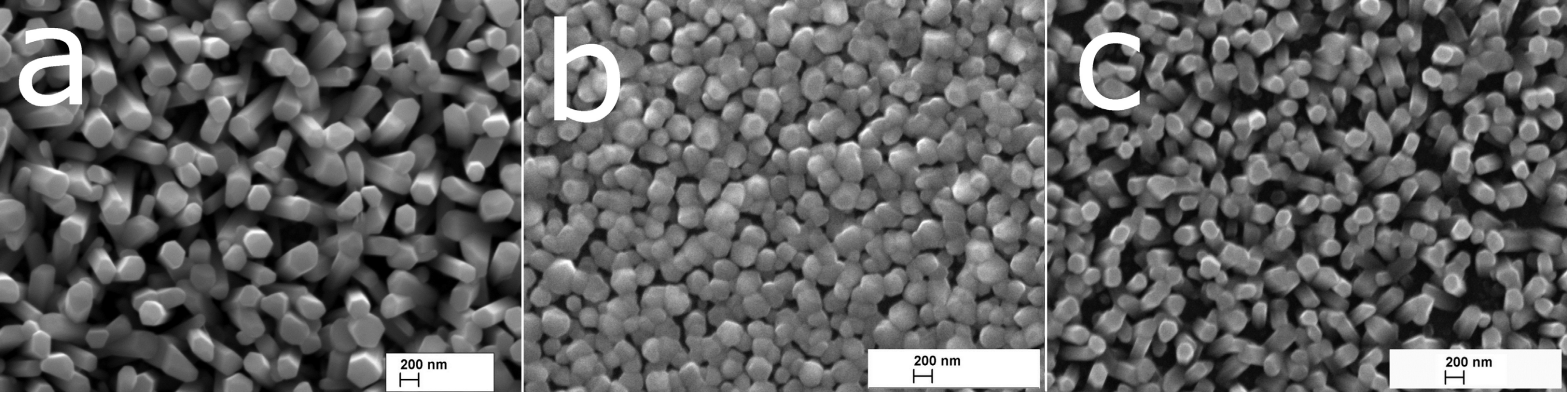
**SEM images of ZnO nanorod layers deposited by chemical spray pyrolysis**. The layers (**a**, **b**, **c**) were deposited onto ITO/SGL substrate at growth temperature of 550°C (a) or 480°C (b, c) using aqueous (a, b) or alcoholic solutions (c) at similar spray rate (2.2 ml/min).

SEM surface images of ZnO_NRL _grown onto ITO/SGL substrate are presented as Figure 3. The ZnO_NRL _deposited from aqueous solution of ZnCl_2 _(0.1 mol/L) at *T*_G _= 550°C is composed of separately standing ZnO crystals with a diameter of 100-300 nm and length of ca. 800 nm, see Figure 3a. When grown at a lower temperature of *T*_G _= 480°C using aqueous solutions, the rod-like shape of the crystals is not well-developed (Figure 3b) as also shown in our earlier study [22]. However, the deposition of an alcoholic solution at *T*_G _= 480°C results in well-developed hexagonal rods with a diameter of ca. 100-200 nm and length of ca. 700-800 nm (see Figure 3c). This result indicates that the spray of an alcoholic solution allows to obtain ZnO_NRL _composed of rods with high aspect ratio at lower substrate temperature compared to the spray of aqueous solution.

**Figure 4:**
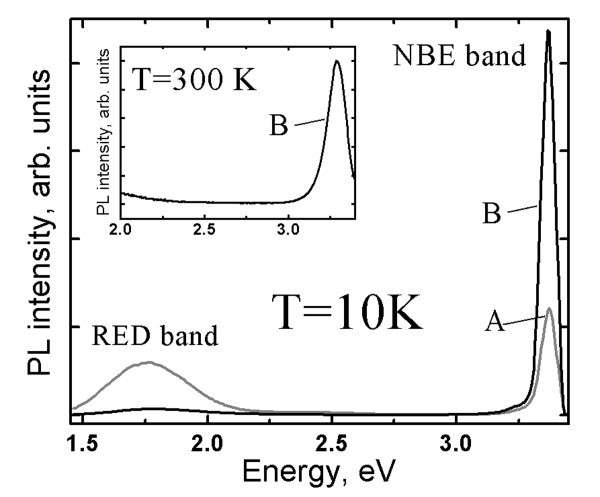
**Photoluminescence spectra at 10 K of the spray pyrolysis-deposited ZnO nanorod layers**. The layers were deposited from aqueous solution onto SGL or ITO/SGL substrates, illustrated by spectrum A and B, respectively. The growth temperature (550°C) and spray rate (6.2 ml/min) were kept similar. The inset shows the photoluminescence spectrum at 300 K in region of 2.0-3.4 eV, characteristic of ZnO nanorods deposited onto ITO/SGL substrate at temperature of 550°C.

The PL spectra of the best ZnO_NRL _samples show a dominant near band edge (NBE) emission at *T *= 10 K and at *T *= 300 K, see Figure 4 (further discussion in Effect of the substrate on PL properties of ZnO_NRL_, Effect of the growth temperature on PL properties of ZnO_NRL_, Effect of the spray rate on PL properties of ZnO_NRL_, and Effect of solvent type on PL properties of ZnO_NRL _sections). The NBE peak positions were determined by fitting the spectra with the Lorentz distribution using the Fityk curve-fitting freeware. The NBE peak measured at *T *= 300 K is due to free exciton transition. High-resolution PL measurements at *T *= 10 K reveal four transitions due to bound excitons: a prevailing exciton peak at 3.358-3.360 eV, two other exciton peaks at 3.363 eV and 3.368-3.370 eV, a peak of the two electron transition at 3.334-3.335 eV and the free exciton peak at 3.378 eV [26,29-32]. The exciton peak at ca. 3.360 eV, which is attributed to a donor-related transition by many authors [26,29-32], is present as the dominant transition, irrespective of the deposition conditions.

### Effect of the substrate on PL properties of ZnO_NRL_

The PL spectra of ZnO_NRL _deposited at *T*_G _of 550°C onto the SGL and the ITO/SGL substrates, are presented as Figure 4. We can see a drastic reduction of the red band intensity at 10 K in case of ZnO_NRL _on ITO/SGL substrate. In addition, the green PL emission band in the region of 2.2-2.8 eV is not present at *T *= 10 K and at *T *= 300 K in case of the ITO/SGL substrate used. This could be expected because strong red and green bands do not tend to co-exist [26,33].

The broad and structureless green luminescent band is mostly reported as an intrinsic defect level due to V_O _[34,35] or V_Zn _[36] or both, acting as a donor-deep-acceptor pair [37]. Although controversial, the band is most likely a native complex defect of zinc vacancy involving Zn_i_, O_Zn_, and V_O _[26]. The origin of another visible Gaussian band, the red band in region of 1.5-2.0 eV, is attributed to interstitial-type defects like Zn_i_, O_i _[15,18] while PL bands between the red and the green band are commonly attributed to O_i _[18,38-40]. Without a comprehensive study additional to PL spectroscopy, the defect identification remains speculative [15].

At room temperature (*T *= 300 K), for the ZnO_NRL _grown at 550°C onto ITO/SGL, no green emission is detected, see Figure 4 inset. Therefore, we conclude that ZnO_NRL _with a lower content of defects can be deposited using the ITO instead of SGL substrates, however, such a growth mechanism of the ZnO_NRL _onto the ITO remains unclear. It may be speculated that a more homogenious lateral heat distribution may be responsible for the significant improvement of the NBE to red band intensity ratio (I_NBE_/I_RED_) in the case of using the ITO/SGL substrate for deposition of ZnO_NR_. Another speculation may be that ITO acts as a barrier, restraining the diffusion of some elements, *e.g.*, sodium from the SGL glass during high-temperature spray process as it has been recorded by X-ray photoelectron spectroscopy for the CSP-deposited CuInS_2 _films [41]. In such a case, borosilicate glass could be a suitable replacement for the SGL substrate. Whatever the reason might be, ITO-covered substrates are preferred in order to achieve ZnO_NRL _with high crystal quality during the CSP process. This observation is of significant importance due to the fact that for many electronic applications including solar cells, a conductive and transparent material, such as ITO, is required as the substrate material.

### Effect of the growth temperature on PL properties of ZnO_NRL_

**Figure 5:**
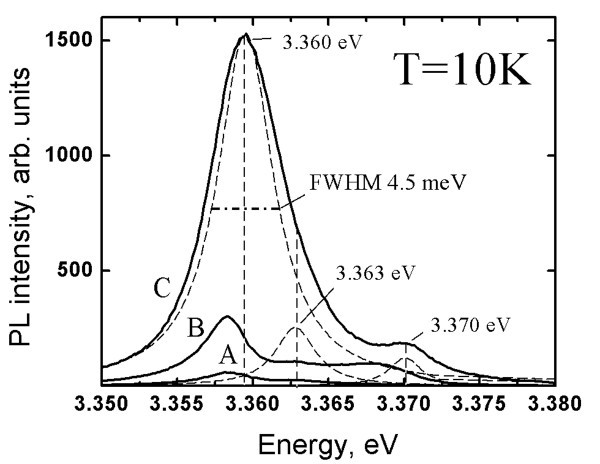
**Excitonic photoluminescence spectra at 10 K of ZnO nanorod layers deposited at different growth temperatures**. The growth temperature is kept at 480°C, 530°C, or 550°C, illustrated by spectrum A, B, and C, respectively. The ZnO nanorod layers are deposited onto ITO/SGL substrates from aqueous solution at similar spray rate (2.2 ml/min). Spectrum C is shown with fitting results, Raman spectrum of the corresponding ZnO nanorod sample is presented in Figure 1 and the SEM image is presented in Figure 3a. The SEM image of the sample corresponding to spectrum A is presented in Figure 3b.

From our previous study, it is known that the increase of *T*_G _from 480°C up to 550°C minimizes the intensity of the green PL band at 300 K [25]. The spectra recorded at *T *= 10 K (Figure 5) reveal that an increase of *T*_G _from 480°C up to 550°C results in a PL spectra with significantly increased excitonic intensity, showing FWHM of 4.5 meV for ZnO_NRL _grown at *T*_G _= 550°C onto ITO/SGL substrate. Such low values of FWHM (4.5 meV) can be correlated to a very low concentration of defects [42]. In addition, the increase of the growth temperature of ZnO_NRL _results in an increase of the ratio of NBE to red band intensity (the red band is not shown in Figure 5). The I_NBE_/I_RED _emission ratio increases as follows: 34, 39, and 160, illustrated by spectrum A, B, and C (in Figure 5), respectively. This is an indication of significant change in the defect composition in the ZnO_NRL _and an increase of the crystal quality due to an increase of the growth temperature of ZnO_NRL_. According to SEM study, the characteristic morphology of the samples changes with different *T*_G_. The SEM image in Figure 3a is a characteristic of ZnO_NRL _grown at *T*_G _= 550°C. A significantly lower surface-to-volume area (no quantitative calculations were made) could be estimated from Figure 3b, presenting the SEM image of ZnO_NRL _deposited at *T*_G _= 480°C. At the same time, an increase of the excitonic PL band was registered without a decrease of the FWHM of the fitted peaks (Figure 5, spectra A-C). Therefore, the increase of the intensity of the excitonic band could be due to a higher surface-to-volume ratio of the ZnO_NRL_, in addition to an increased crystal quality. A similar effect was described for the electrochemically synthesized ZnO nanowires [20].

In connection with the results in the Effect of the substrate on PL properties of ZnO_NRL _section, higher *T*_G _of 550°C and the ITO/SGL substrates are preferred for the deposition of ZnO_NRL _in order to achieve high optical quality by the CSP method. The best samples were grown at 550°C onto ITO/SGL substrate, showing NBE emission only. Although, no significant reduction in FWHM is observed while using a higher growth temperature of 550°C, an apparent shift of the main NBE peak at approximately 3.360 eV towards higher energy could be an indicator of additional transitions. The high ratio of I_NBE _to I_VISIBLE _emissions alone, although widely reported as an indication of good [43] or even excellent optical quality [7,44], may not be enough to characterize the purity of ZnO_NRL _[15,43]. However, the resolvable fine structure of the excitonic PL emission could be taken as an indication of a relatively high crystal quality of the CSP deposited ZnO_NRL_.

### Effect of the spray rate on PL properties of ZnO_NRL_

**Figure 6:**
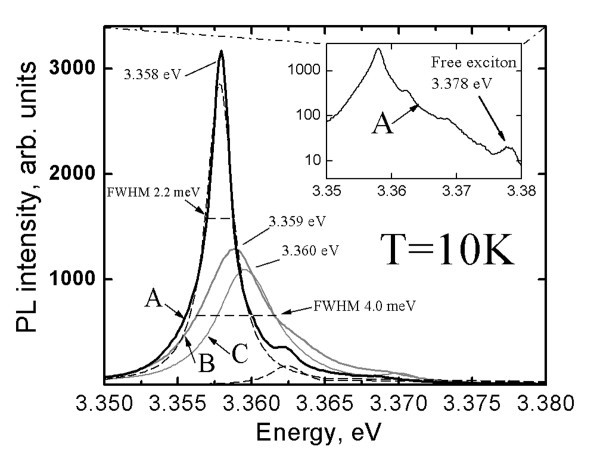
**Excitonic photoluminescence spectra at *T *= 10 K of ZnO nanorod layers deposited at different spray rates**. The layers are deposited from aqueous solutions onto ITO/SGL substrates at 550°C with spray rate of 1.2, 2.2, and 6.2 ml/min, illustrated by spectrum A, B, and C, respectively. Spectrum A is shown with fitting results. The inset in the Figure 6 shows spectrum A in logarithmic scale.

The effect of the variation in the spray rate on PL properties of ZnO_NRL _is presented as Figure 6. A decrease in the spray rate from 6.2 ml/min down to 1.2 ml/min increases the intensity of the exciton peak at 3.358-3.360 eV.

The rise in the intensity of the peaks could be correlated to a longer deposition period, hence, a lower growth rate. In addition, the FWHM of the main peak decreases from 4.0 meV down to 2.2 meV, accompanied by a small shift from 3.360 eV to 3.358 eV in the exciton peak position. A shift in the position of a defect-bound exciton is a clear indication of a change in the type of the dominant impurity in the ZnO_NRL_, while a smaller FWHM is a sign of increased crystal quality. For comparison, peak widths of 6-9 meV and 4.7 meV are reported for ZnO_NRL _grown by hydrothermal method with post-deposition annealing [15] and thermal evaporation and vapor-phase transport [7], respectively. A FWHM of 1 meV is characteristic of the excitonic peak at 3.360 eV reported for a single crystal MOCVD-deposited ZnO_NRL_, measured at 3.4 K [31]. The PL intensity in a log-scale reveals a free exciton peak at 3.378 eV appearing only at a spray rate of 1.2 ml/min (see Figure 6 inset), providing confirmation that a low defect density is obtained in ZnO_NRL _[45]. Thus, in addition to high growth temperatures, low spray rates are preferred for CSP deposition of ZnO_NRL _in case aqueous solutions are used.

### Effect of solvent type on PL properties of ZnO_NRL_

**Figure 7:**
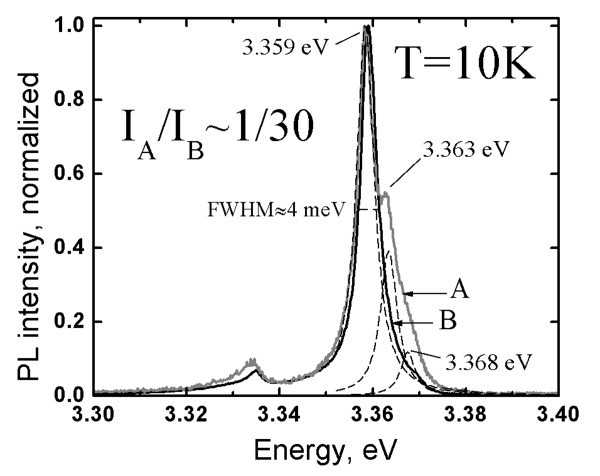
**Normalized photoluminescence spectra of ZnO nanorod layers deposited using different solvents**. The layers are deposited onto ITO/SGL substrate at 480°C from aqueous or alcoholic solution, illustrated by spectrum A and B, respectively. Spray rate is kept similar (2.2 ml/min). Spectrum A is shown with fitting results. SEM image of the sample corresponding to spectrum B is presented in Figure 3c.

The effect of the use of different solvents on the PL properties of ZnO_NRL _is presented as Figure 7. An exciton peak at 3.363 eV is observed only when aqueous solutions are used for deposition of ZnO_NRL_. A shallow donor impurity corresponding to an excitonic peak at approximately 3.363 eV has been correlated to hydrogen impurity by many authors, see for example [29]. However, we can present no proof for hydrogen incorporation in this study, thus, it will not be considered further.

In case the alcoholic solvent is used, the excitonic band intensity increases approximately 30 times. The PL intensity ratio I_NBE_/I_RED _increases from 34 in case of aqueous up to 294 in case alcoholic solution is used. This observation could be explained taking into account a similar observation from the effect of the *T*_G _(Effect of the growth temperature on PL properties of ZnO_NRL _section). The use of an alcoholic solution results in a smaller spray droplet size, leading to a more homogeneous distribution of the sprayed solution over the substrate, compared to an aqueous solution. In addition to a smaller droplet size, the high volatility of the alcoholic solvent is very likely to cause a lower rate of the substrate cooling. Thus, a higher value of the effective growth temperature is expected in the reaction zone of pyrolysis, resulting in a somewhat higher growth temperature than the stated *T*_G_. This can be discussed in the following.

Due to the fact that the PL spectrum A in Figure 7 coincides with spectrum A in Figure 5, we can see that the use of an alcoholic solution instead of an aqueous solution increases the NBE band intensity (approximately × 30) in ZnO_NRL _deposited at 480°C (Figure 7). Similar effect has been observed while increasing the *T*_G _from 480°C up to 550°C (Figure 5). In Effect of the growth temperature on PL properties of ZnO_NRL _section, we tentatively attributed this effect to an increased surface-to-volume ratio of the ZnO_NRL_. Similarly, the ZnO_NRL _grown at 480°C from alcoholic solution (see SEM image in Figure 3c) shows a significantly increased surface-to-volume ratio compared to ZnO_NRL _grown at 480°C from aqueous solution (see Figure 3b). Therefore, one possible cause of a higher NBE intensity of spectrum B compared to spectrum A in Figure 7, could be a higher surface-to-volume ratio, in addition to improved crystal quality.

Although the density of rods and the aspect ratio is known to increase when deposited from alcoholic solutions [46], the effect of the temperature cannot be overlooked either in the sense of an increased NBE intensity. As a result, it can be concluded that a lower *T*_G _or a higher spray rate is acceptable for the CSP deposition of the ZnO_NRL _in case an alcoholic solution is used, compared to the *T*_G _and spray rates used for aqueous solutions (Effect of the growth temperature on PL properties of ZnO_NRL _and Effect of the spray rate on PL properties of ZnO_NRL _sections). The use of an alcoholic instead of aqueous solution could be applied in order to achieve a higher surface-to-volume ratio at a similar *T*_G_. It must be pointed out, however, that the PL of the ZnO_NRL _deposited at a low spray rate (1.2 ml/min) from aqueous solution was superior to all considered samples in sense of narrow NBE peaks (Effect of the spray rate on PL properties of ZnO_NRL _section).

## Conclusions

We report photoluminescence properties in as-deposited ZnO nanorod layers (ZnO_NRL_), grown via fast and low-cost pneumatic CSP method in air. The results indicate that a good crystal quality can be achieved, while ZnO_NRL _with the best optical quality were deposited at a growth temperature of 550°C using ITO/SGL substrate. No green or red emission was detected for these samples, measured at 10 K. In addition, green emission is absent at 300 K for the best samples. The prevalence of the bound exciton transitions suggests a low defect density in our best ZnO_NRL_. Low spray rates (1.2 ml/min) are recommended in case aqueous solution is sprayed, otherwise alcoholic solution is preferred for the deposition of ZnO_NRL_. Lower substrate temperature is acceptable in case alcoholic solution is used, in order to achieve comparable crystal quality, compared to the use of aqueous solution. At *T *= 10 K, fitted excitonic peak widths (FWHM) are 2-5 meV. For comparison, peak widths of 6-9 meV are reported for ZnO_NRL _grown by hydrothermal method with post-deposition annealing [15]. These results indicate that the quality of the spray-deposited ZnO_NRL _is superior compared to other solution-based methods in the sense of photoluminescence properties. The results of this study imply that the spray-deposited ZnO_NRL _is sufficient for the use as a transparent window layer in an ETA solar cell and for applications to other low-cost photonic devices at room temperature.

## Competing interests

The authors declare that they have no competing interests.

## Authors' contributions

EK carried out photoluminescence experiments, analysis, and interpretation of experimental results and writing of major part of paper, TR carried out photoluminescence experiments, TD designed and deposited ZnO nanorods, JK provided valuable theoretical discussions, AM provided valuable experimental discussions, VM carried out SEM study, MK designed the study, revised the manuscript, provided motivation and discussions. All authors read and approved the final version of the manuscript.
